# Supplementary material for: Tuning the Envelope Structure of Enzyme Nanoreactors for In Vivo Detoxification of Organophosphates
Source: Int J Mol Sci. 2023 Oct 30;24(21):15756. doi: 10.3390/ijms242115756 (PMC10649860; doi:10.3390/ijms242115756)
Supplement: Supplementary file 1 [file ijms-24-15756-s001.zip › ijms-2642600-supplementary.pdf]

Supplementary Material

for

Tuning the envelope structure of enzyme nanoreactors for *in vivo* detoxification of organophosphates

Tatiana Pashirova [1]*, Zukhra Shaihutdinova [1,2], Dmitry Tatarinov [1], Milana Mansurova [2], Renata Kazakova [2], Andrei Bogdanov [1], Eric Chabrière [3,4], Pauline Jacquet [3], David Daudé [3], Almaz Akhunzianov [2], Regina Miftakhova [2], Patrick Masson [2]*

[1] Arbuzov Institute of Organic and Physical Chemistry, FRC Kazan Scientific Center, Russian Academy of Sciences, Arbuzov Str., 8, Kazan, 420088, Russia

[2] Institute of Fundamental Medicine and Biology, Kazan Federal University, 420008 Kazan, Russia

[3] Gene&GreenTK, 19–21 Boulevard Jean Moulin, 13005 Marseille, France

[4] IRD, APHM, MEPHI, IHU-Méditerranée Infection, Aix Marseille Université, 19–21 Boulevard Jean Moulin, Moulin, 13005 Marseille, France

* Correspondence: tatyana_pashirova@mail.ru or pashirova@iopc.ru (T.P.);

pym.masson@free.fr or pmasson@kpfu.ru (P.M.)

Contents

FigureS1. ^{1}H NMR spectra of mPEGSAc-2000 S3

Figure S2. ^{1}H NMR spectra of copolymer **1a** S4

Figure S3. ^{13}C NMR spectra of copolymer**1a** S5

Figure S4. IR spectra of copolymer **1a** S6

Figure S5. ^{1}H NMR spectra of copolymer **1b** S7

Figure S6. ^{13}C NMR spectra of copolymer **1b** S8

Figure S7a. IR spectra of copolymer **1b** S9

Figure S7b. Comparison of IR spectra of copolymer **1a** and **1b** S10

Figure S8. Screen shot of empty nanoreactors distribution based on **1a** copolymer S11

Figure S9. Screen shot of empty nanoreactors distribution based on **1b** copolymer S12

Figure S10. TEM Images of empty nanoreactors based on **1a** S13

Figure S11. TEM Images of empty nanoreactors based on **1b** S14

Figure S12. UV Absorbance spectra of pNp for EE of nanoreactors S15

Figure S13. UV Absorbance spectra of pNp after the neutralization of POX S16

Figure S14. UV Absorbance spectra of enzyme for EE of nanoreactors S17

Figure S15. TEM Images of enzyme-loaded nanoreactors based on 1b S18

Figure S16. Particle size distribution for nanoreators based **1b** S19

Table 1S. *In vitro* stability of nanoreactors based **1b** in blood. S20

Figure S17. UV Absorbance spectra of pNp after the neutralization of POX S21

Table 2S. Prophylaxis and post-exposure treatment of paraoxon S22

Figure S18. Elevated cross maze parameters on the 1st day of experiment S23

Figure S19. Elevated cross maze parameters on the 30th day of experiment. S24

Figure S20. Elevated cross maze parameters on the 1st *vs* 30th days of experiment S25

Figure S21. St's t-test for free enzyme (1) and nanoreactor based on **1b** (2) 1st injection S26

Figure S22. St's t-test for nanoreactor based on **1b** 1st injection and 2nd injection (after 30 days) S27

Figure S23. One Way ANOVA for free enzyme and nanoreactor based on **1b**. S28

Figure S24. Pharmacokinetic profiles in mouse plasma after intravenous injection of free enzyme and enzyme-loaded nanoreactors S29

Table 3S. Pharmacokinetic parameters observed in mouse S30

Figure S25. St's t-test for free enzyme and nanoreactor 2nd injection S31

Figure S26. The ratio of the signals from positive and negative control samples with tested dilution factors S32

Figure S27. The receiver operating characteristic analysis of ELISA S33

Figure S28. The western blot membranes for anti-PTE-IgG from 3 independent experiments S34

Figure S29. UV Absorbance and calibration curve of PTE in Tris buffer S35

Figure S30. Transmittance of polymersomes under centrifugation conditions *vs* time S36

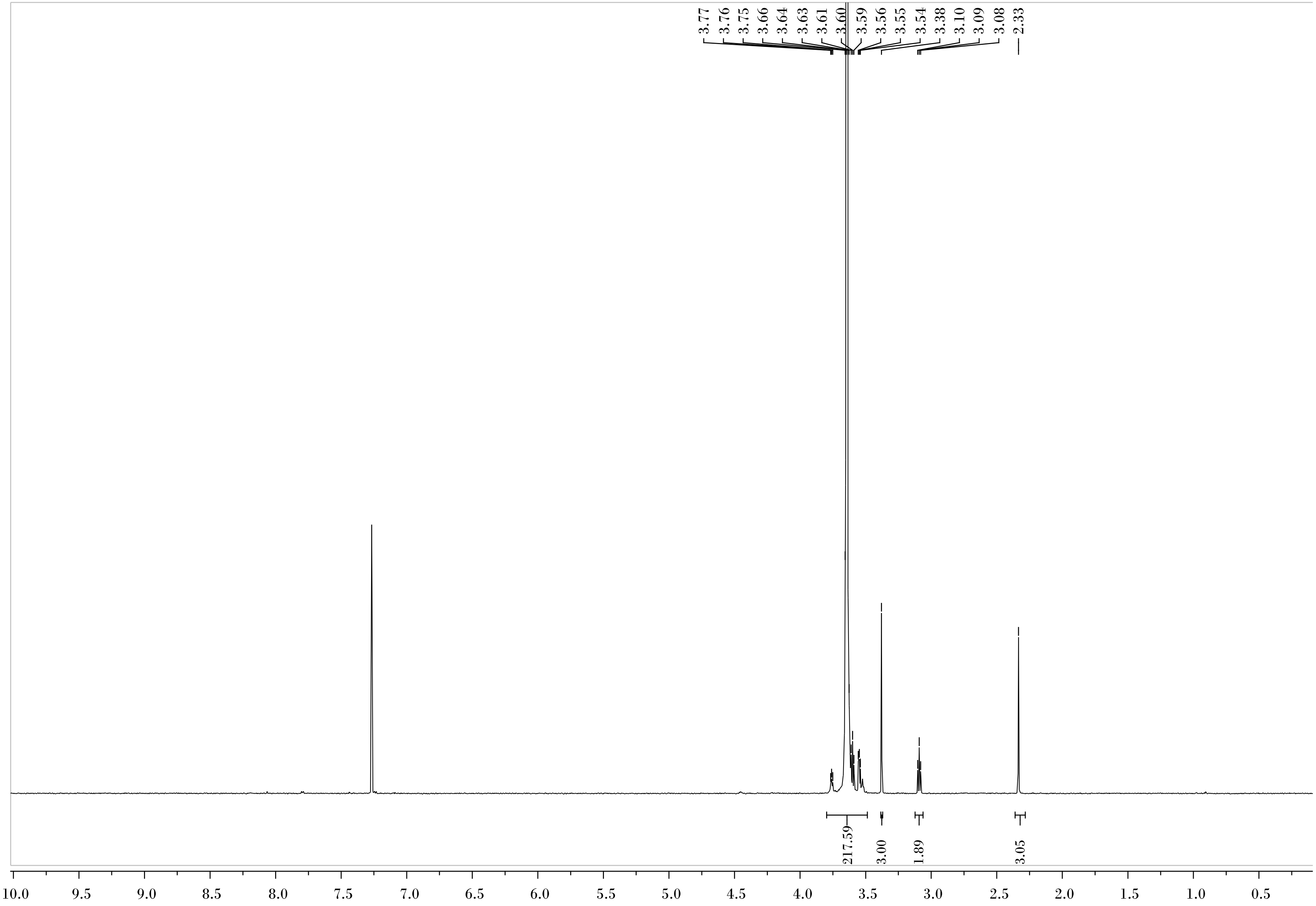

Figure S1. ^{1}H NMR spectra of mPEGSAc-2000

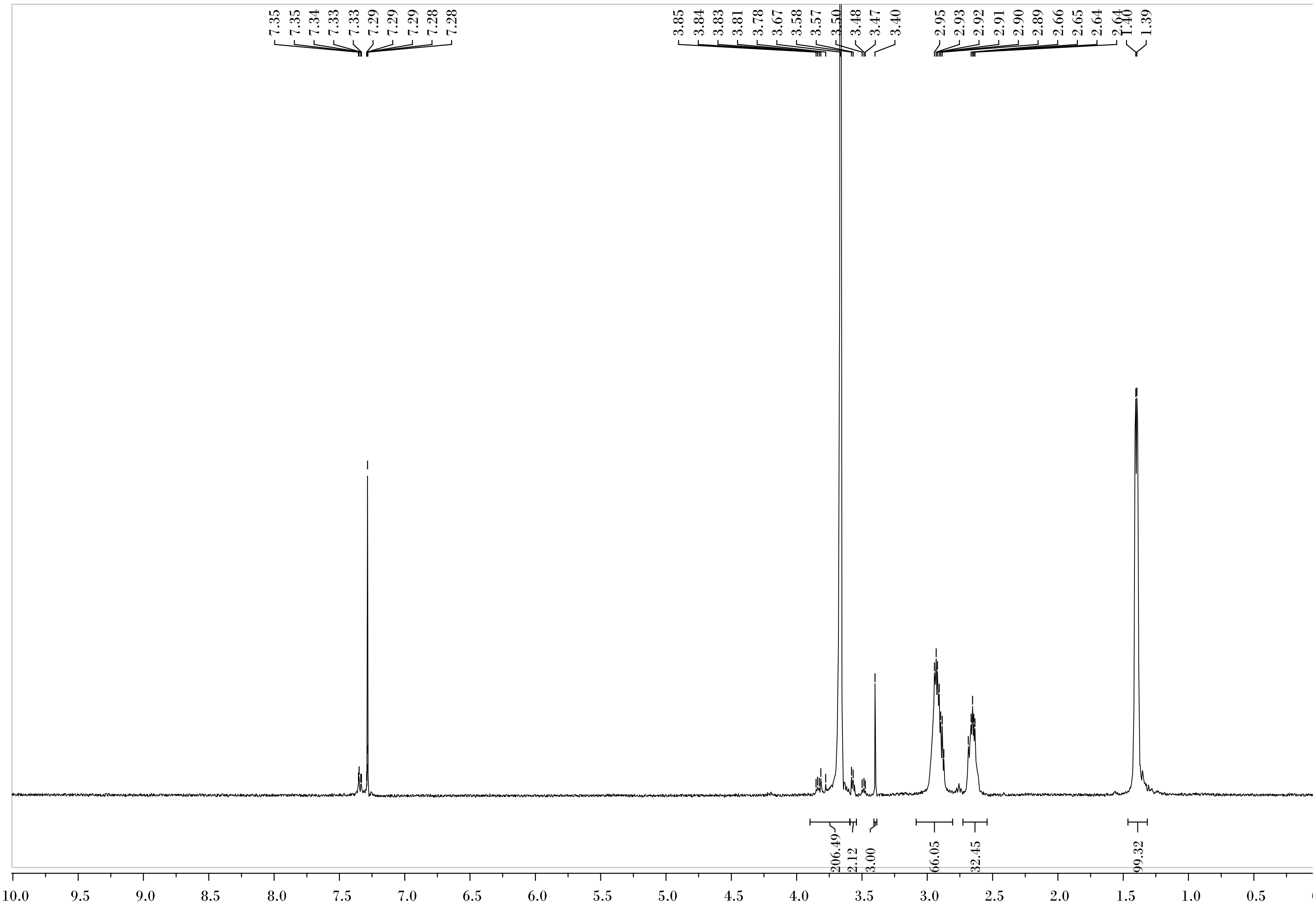

Figure S2. ^{1}H NMR spectra of copolymer **1a**

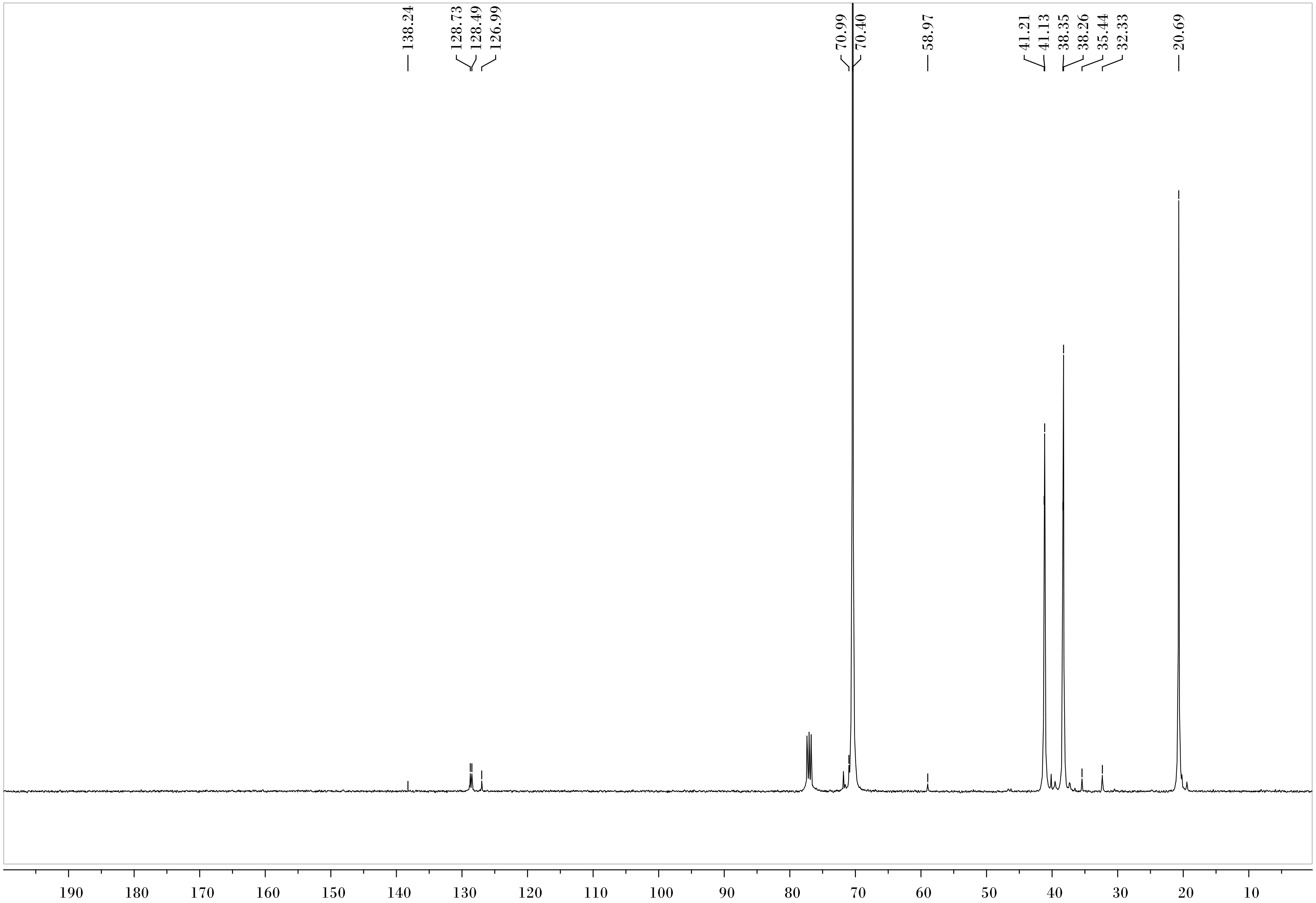

Figure S3 ^{13}C NMR spectra of copolymer **1a**

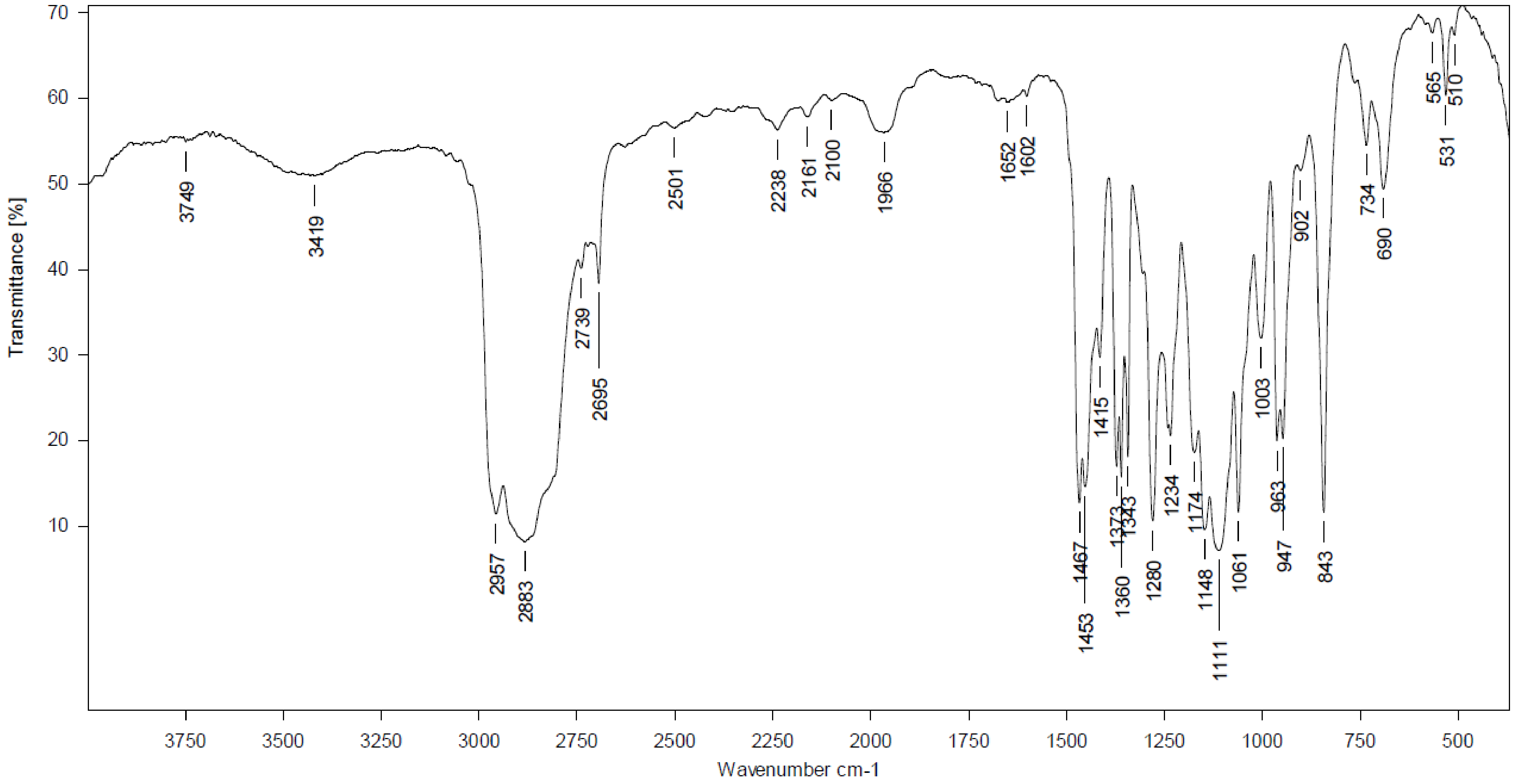

Figure S4. IR spectra of copolymer **1a**

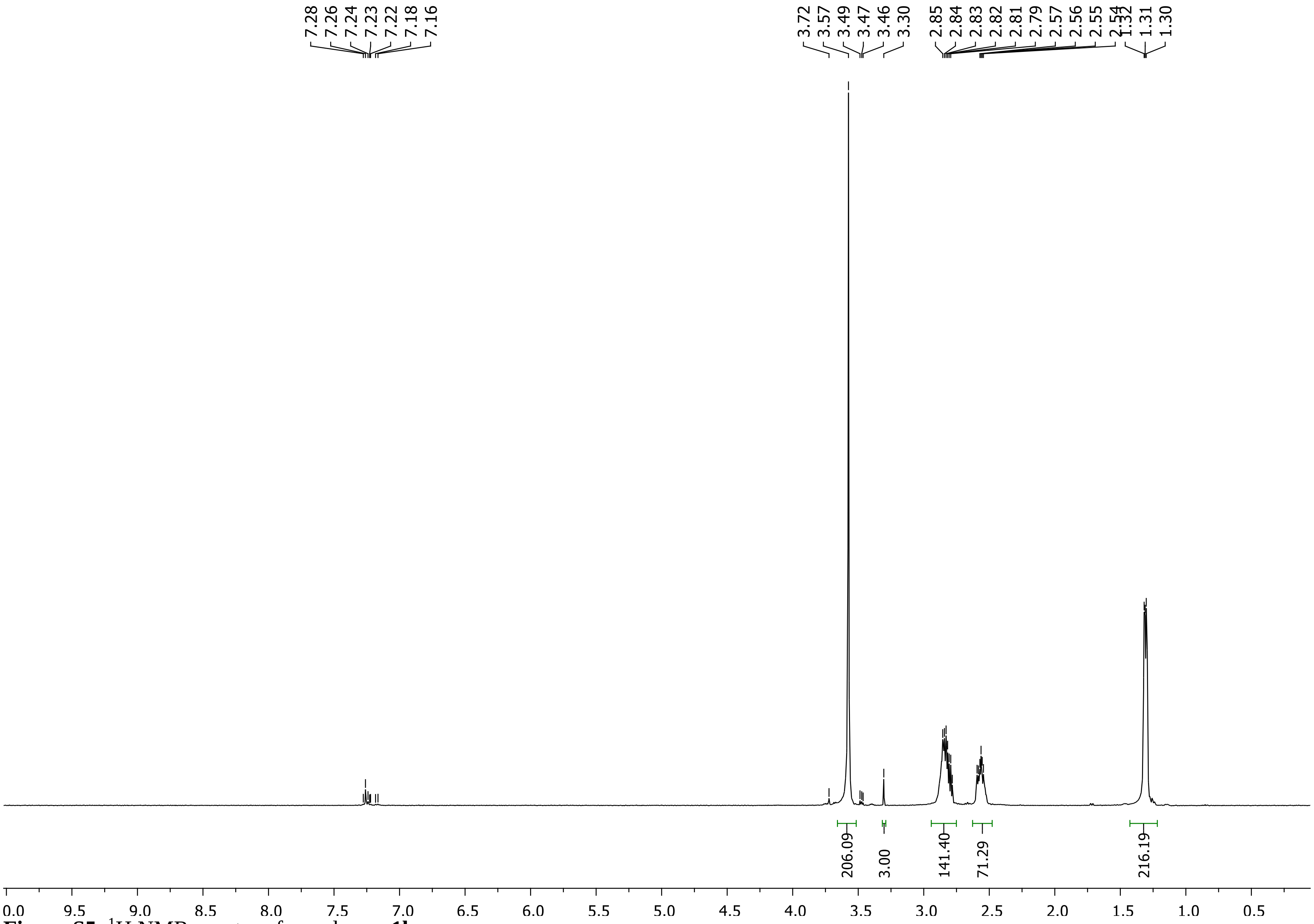

Figure S5. ^{1}H NMR spectra of copolymer **1b**

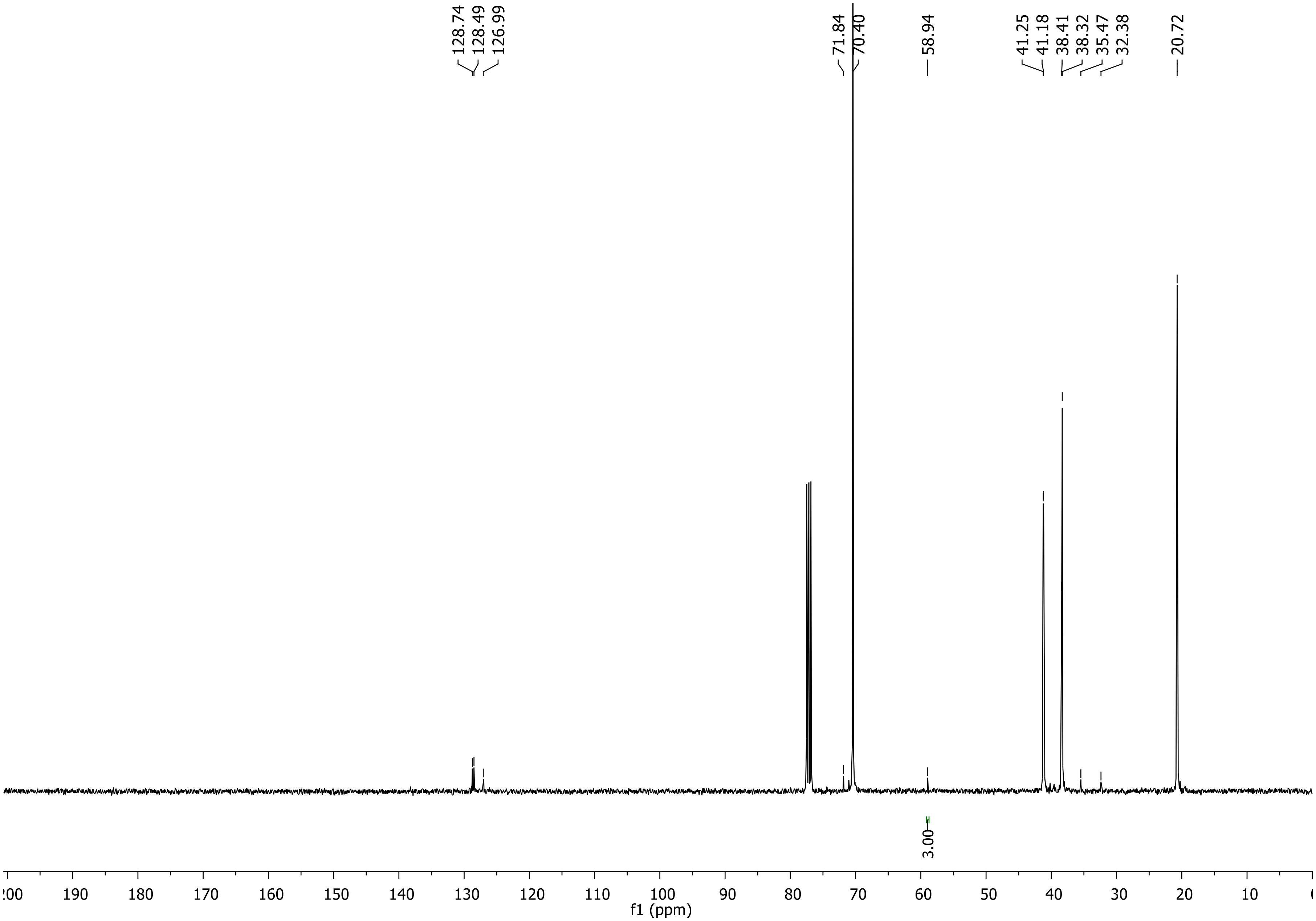

Figure S6. ^{13}C NMR spectra of mPEG-PPS-mPEG copolymer **1b**

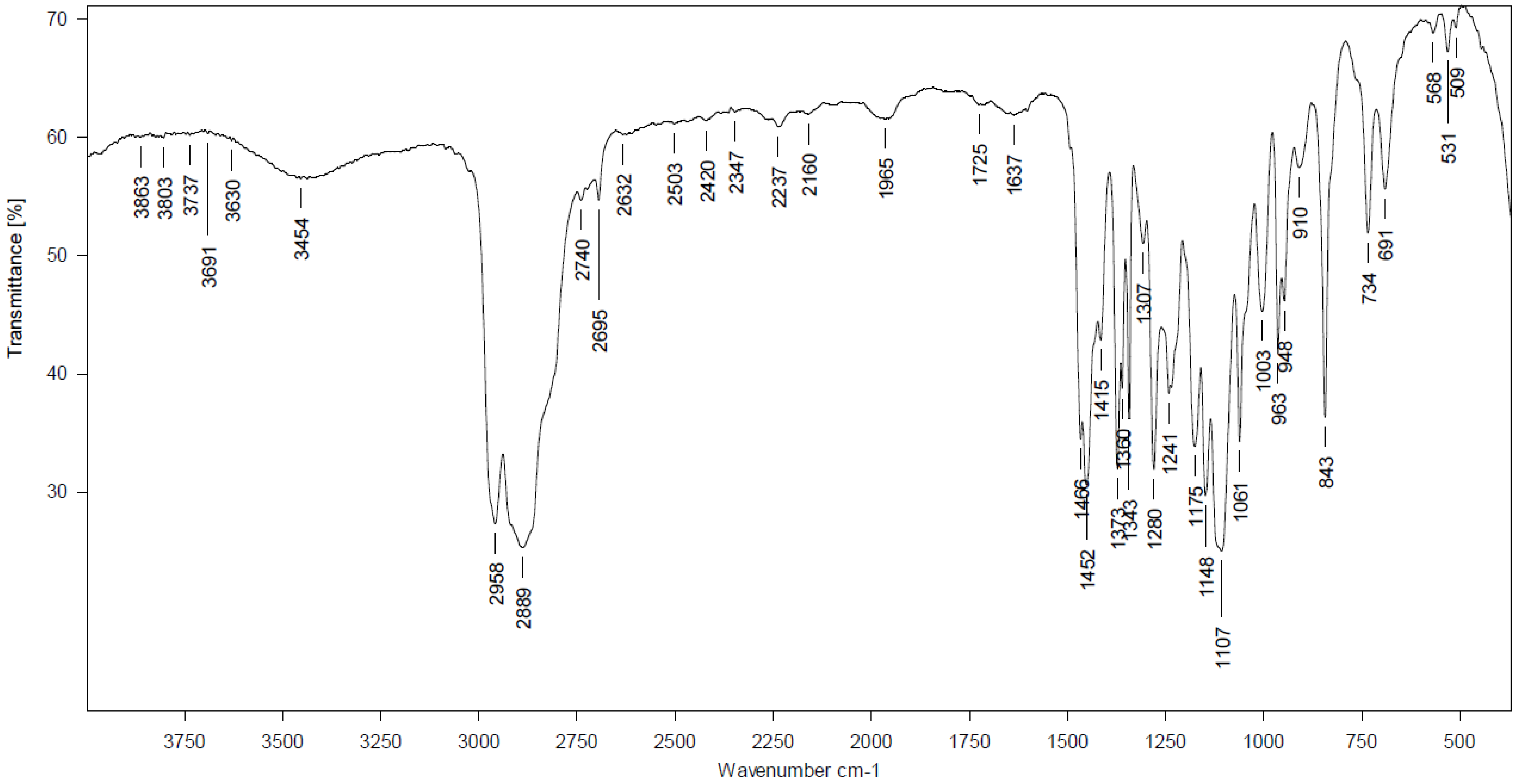

Figure S7a. IR spectra of copolymer **1b**

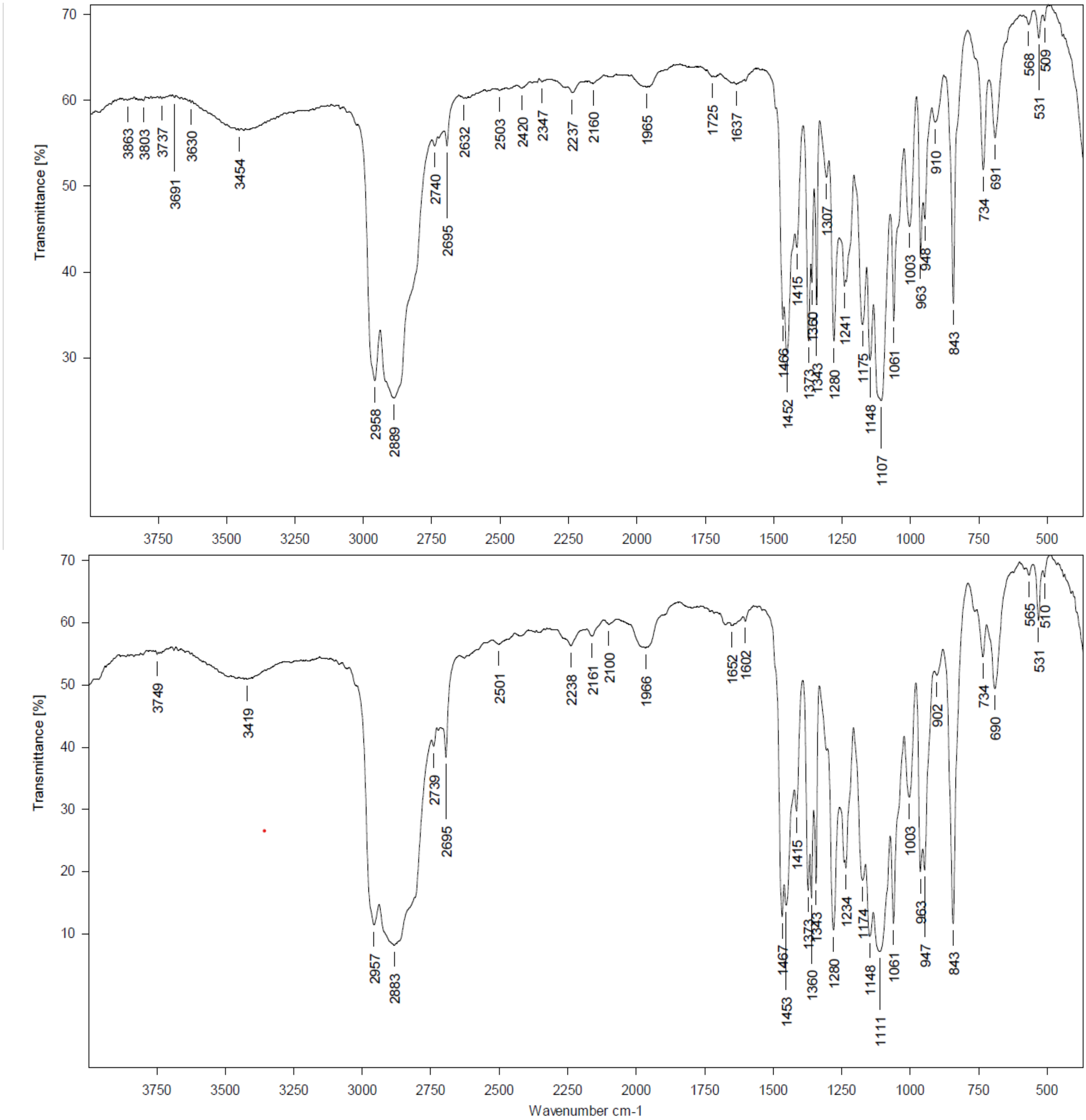

Figure S7b. Compirison of IR spectra of copolymer **1a and 1b**

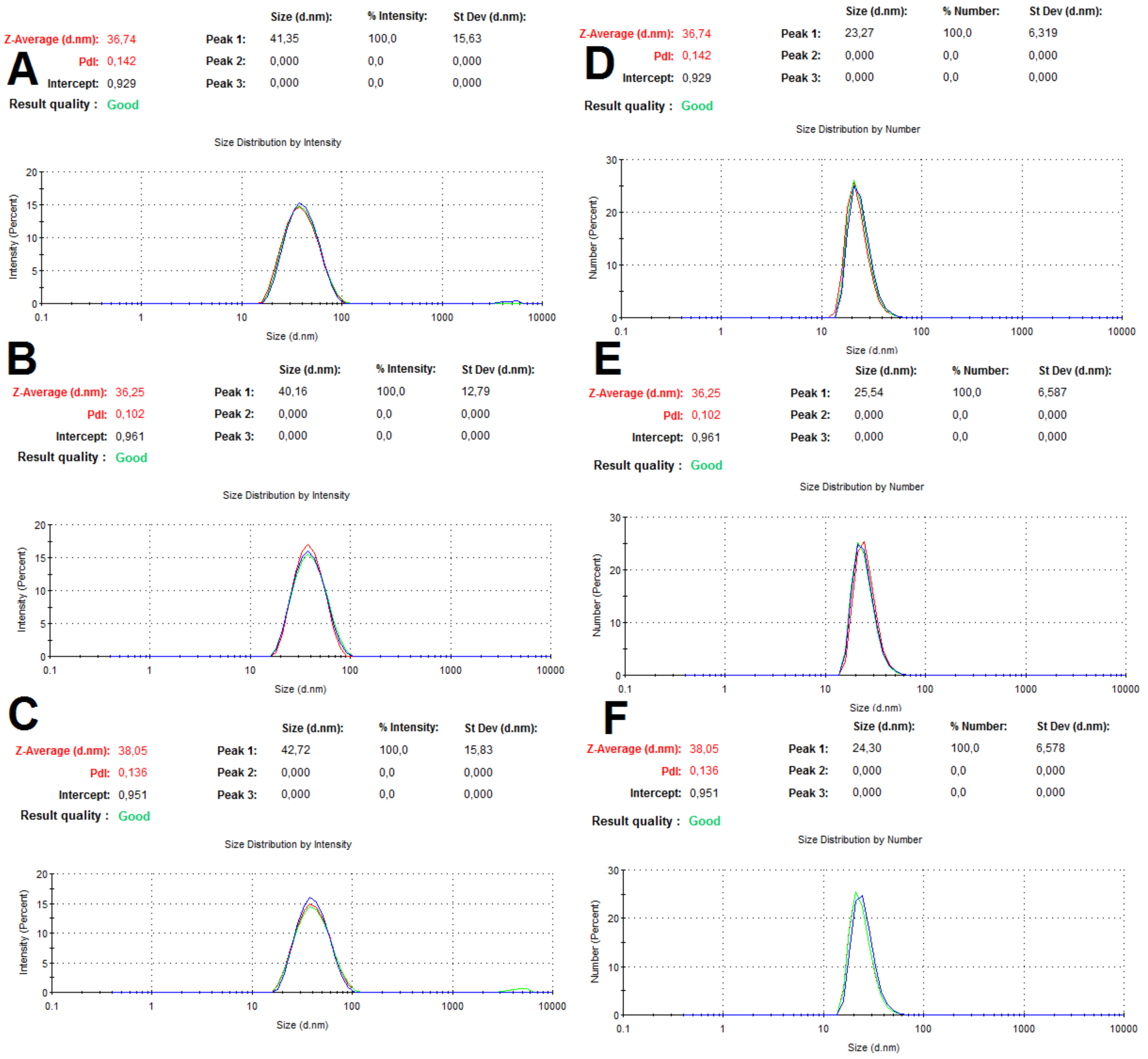

Figure S8. Screen shot of empty nanoreactor size distribution using the intensity(A-C) and number parameters (D-F) based on **1a**, copolymer concentrations 0.1, 0.2, 0.5, 0.75, 1% (wt/wt), 10 mM Tris buffer, pH=7.4, 25ºC.

A

Z-Average (d.nm):	83,91
PdI:	0,157
Intercept:	0,956
Result quality :	Good

	Size (d.nm):	% Intensity:	St Dev (d.nm):
Peak 1:	98,14	100,0	41,56
Peak 2:	0,000	0,0	0,000
Peak 3:	0,000	0,0	0,000

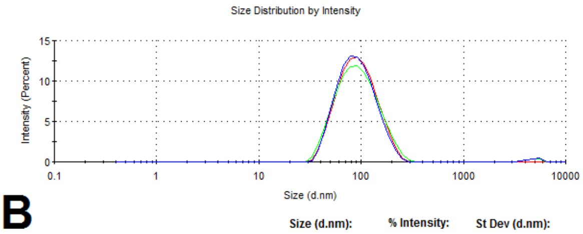

B

Z-Average (d.nm):	80,89
PdI:	0,135
Intercept:	0,968
Result quality :	Good

	Size (d.nm):	% Intensity:	St Dev (d.nm):
Peak 1:	94,92	100,0	39,90
Peak 2:	0,000	0,0	0,000
Peak 3:	0,000	0,0	0,000

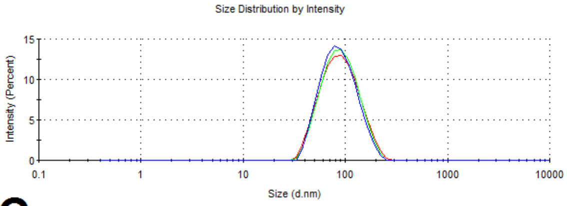

C

Z-Average (d.nm):	92,06
PdI:	0,164
Intercept:	0,934
Result quality :	Good

	Size (d.nm):	% Intensity:	St Dev (d.nm):
Peak 1:	109,1	100,0	48,57
Peak 2:	0,000	0,0	0,000
Peak 3:	0,000	0,0	0,000

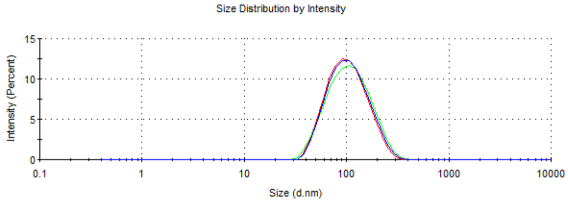

D

Z-Average (d.nm):	110,9
PdI:	0,211
Intercept:	0,964
Result quality :	Good

	Size (d.nm):	% Intensity:	St Dev (d.nm):
Peak 1:	146,0	100,0	81,55
Peak 2:	0,000	0,0	0,000
Peak 3:	0,000	0,0	0,000

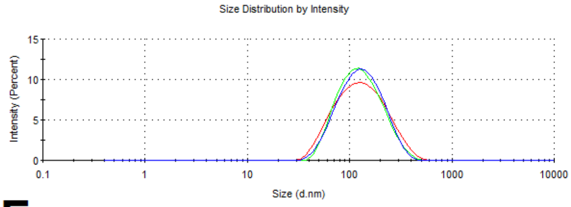

E

Z-Average (d.nm):	106,1
PdI:	0,191
Intercept:	0,951
Result quality :	Good

	Size (d.nm):	% Intensity:	St Dev (d.nm):
Peak 1:	120,3	97,8	51,33
Peak 2:	4553	2,2	849,1
Peak 3:	0,000	0,0	0,000

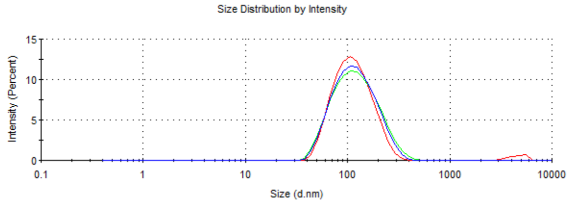

F

Z-Average (d.nm):	83,91
PdI:	0,157
Intercept:	0,956
Result quality :	Good

	Size (d.nm):	% Number:	St Dev (d.nm):
Peak 1:	48,52	100,0	15,03
Peak 2:	0,000	0,0	0,000
Peak 3:	0,000	0,0	0,000

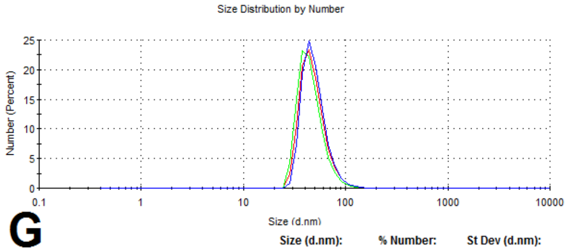

G

Z-Average (d.nm):	80,89
PdI:	0,135
Intercept:	0,968
Result quality :	Good

	Size (d.nm):	% Number:	St Dev (d.nm):
Peak 1:	47,06	100,0	14,48
Peak 2:	0,000	0,0	0,000
Peak 3:	0,000	0,0	0,000

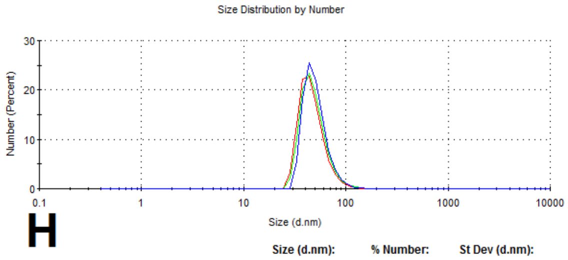

H

Z-Average (d.nm):	92,06
PdI:	0,164
Intercept:	0,934
Result quality :	Good

	Size (d.nm):	% Number:	St Dev (d.nm):
Peak 1:	52,61	100,0	16,49
Peak 2:	0,000	0,0	0,000
Peak 3:	0,000	0,0	0,000

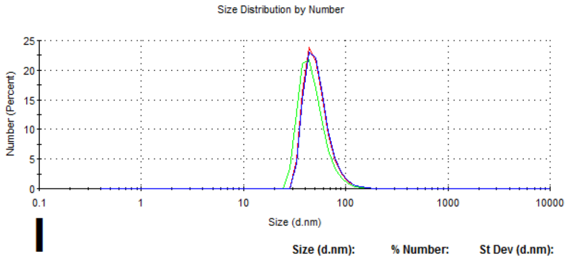

I

Z-Average (d.nm):	110,9
PdI:	0,211
Intercept:	0,964
Result quality :	Good

	Size (d.nm):	% Number:	St Dev (d.nm):
Peak 1:	50,47	100,0	17,99
Peak 2:	0,000	0,0	0,000
Peak 3:	0,000	0,0	0,000

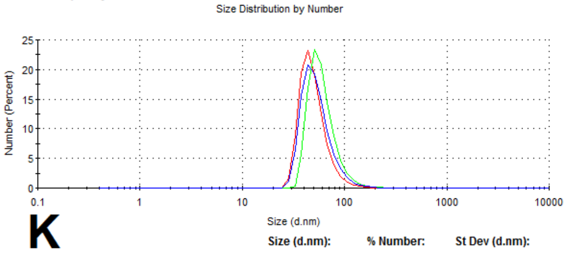

K

Z-Average (d.nm):	106,1
PdI:	0,191
Intercept:	0,951
Result quality :	Good

	Size (d.nm):	% Number:	St Dev (d.nm):
Peak 1:	61,60	100,0	19,53
Peak 2:	0,000	0,0	0,000
Peak 3:	0,000	0,0	0,000

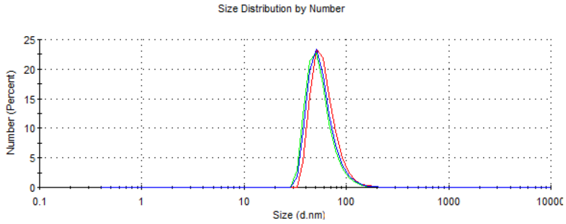

Figure S9. Screen shot of empty nanoreactor size distribution using the intensity(A-E) and number parameters (F-K) based on **1b**, copolymer concentrations 0.1, 0.2, 0.5, 0.75, 1% (wt/wt), 10 mM Tris buffer, pH=7.4, 25ºC.

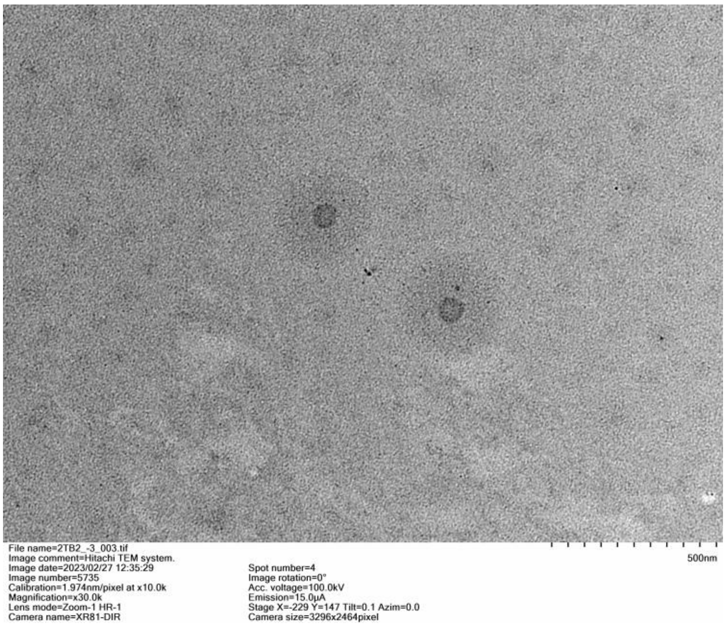

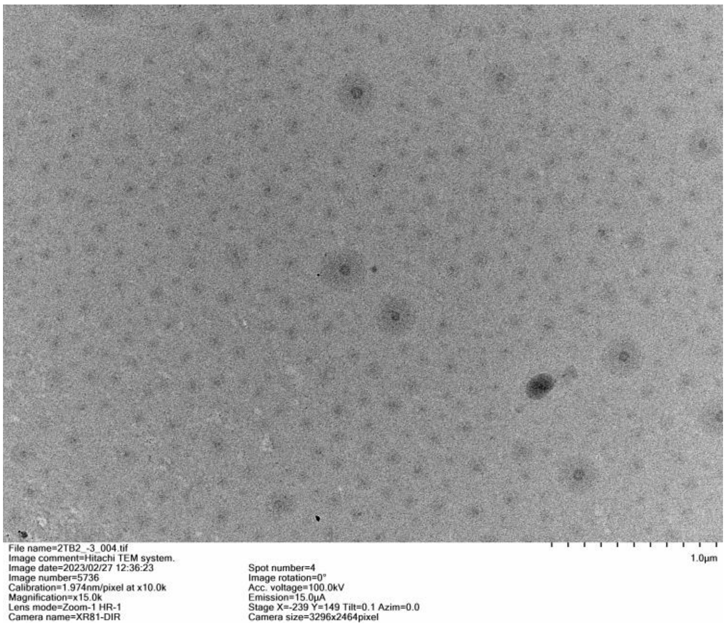

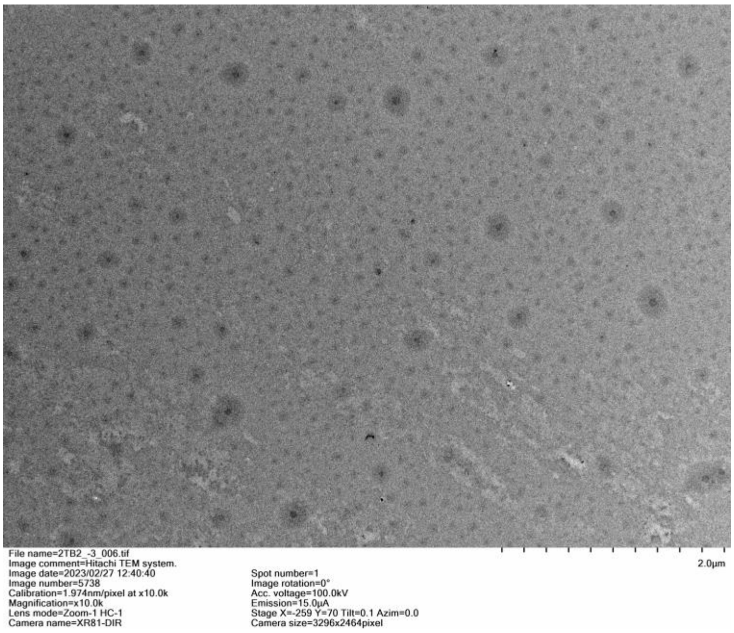

Figure S10. TEM Images of empty nanoreactors based on **1a**, $C_{\mathbf{1a}}$ = 10 μg/mL, Tris-Buffer, pH = 7.4, 25 °C.

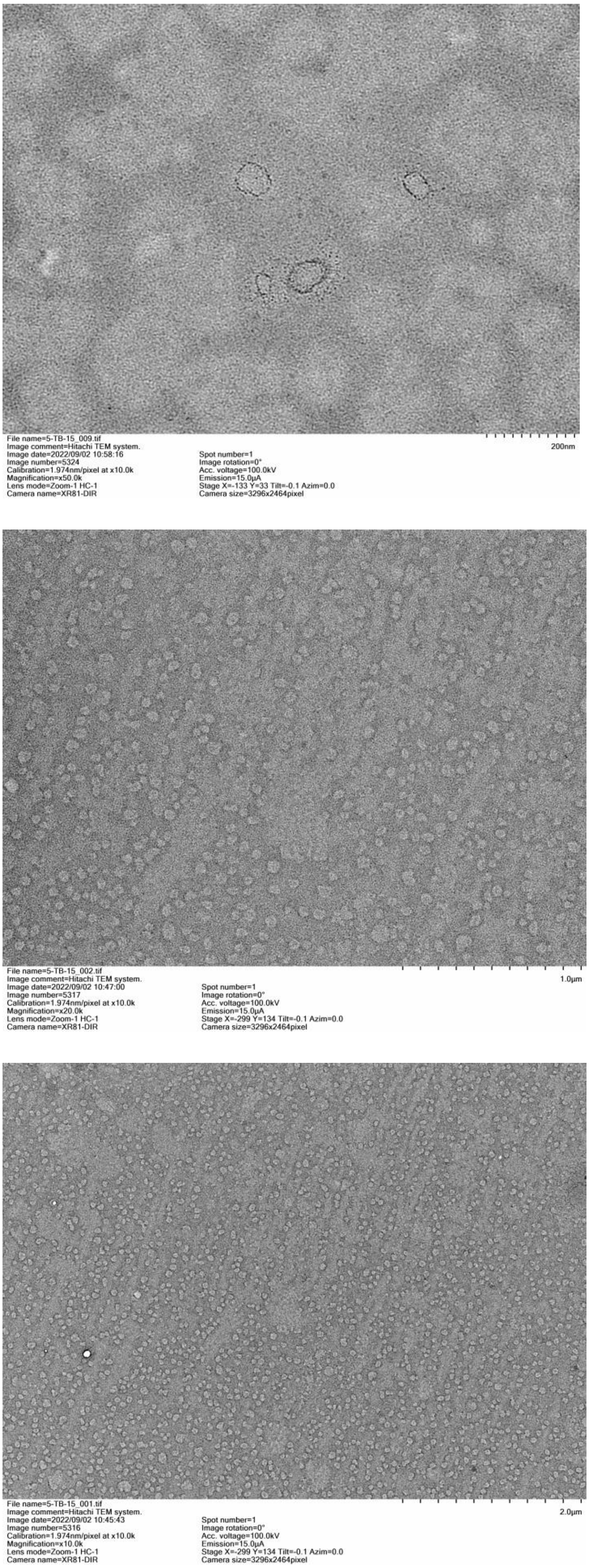

Figure S11. TEM Images of empty nanoreactors based on **1b**, $C_{\mathbf{1b}}$ = 10 μg/mL, Tris-Buffer, pH = 7.4, 25 °C.

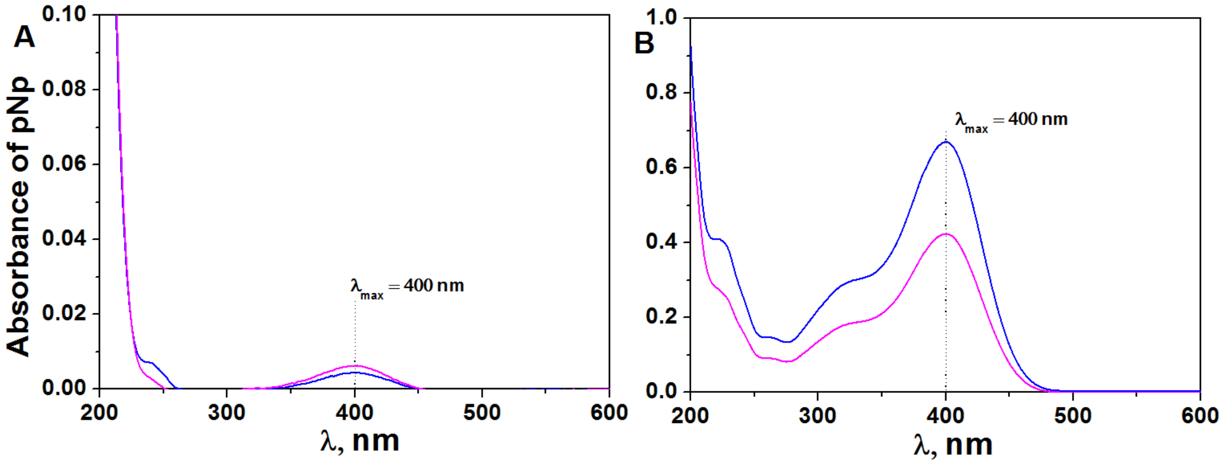

Figure S12. UV Absorbance spectra of pNp for encapsulation efficiency of nanoreactors based on **1b** (A) and **1a** (B), 10mM Tris buffer, pH=7.4, 25°C, $C_{polymers}$ (wt/wt) = 0.5%.

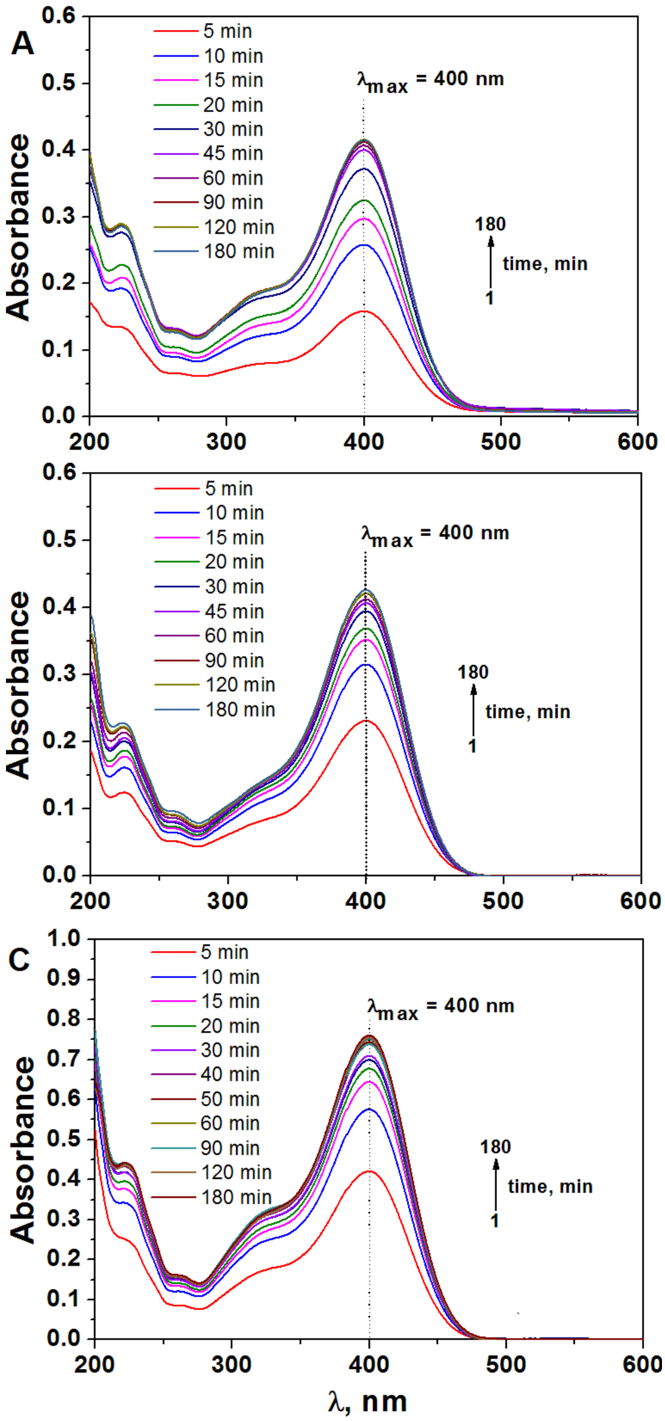

Figure S13. UV Absorbance spectra of pNp from nanoreactors **1a (B)** and **1b (C)**, where control is in the absence of nanoreactors (A), $C_{1a} = C_{1b}$. = 0.5% (wt/wt), C_{pNp} = 0.1% (wt/wt) 37 °C, 10 mM Tris-Buffer, pH = 7.4.

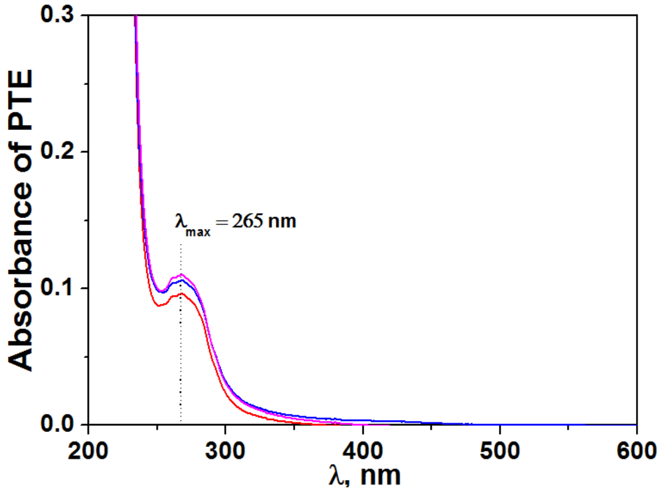

Figure S14. UV Absorbance spectra of enzyme for encapsulation efficiency of nanoreactors, 10mM Tris buffer, pH=7.4, 25°C, C_{1b} (wt/wt) = 0.5%.

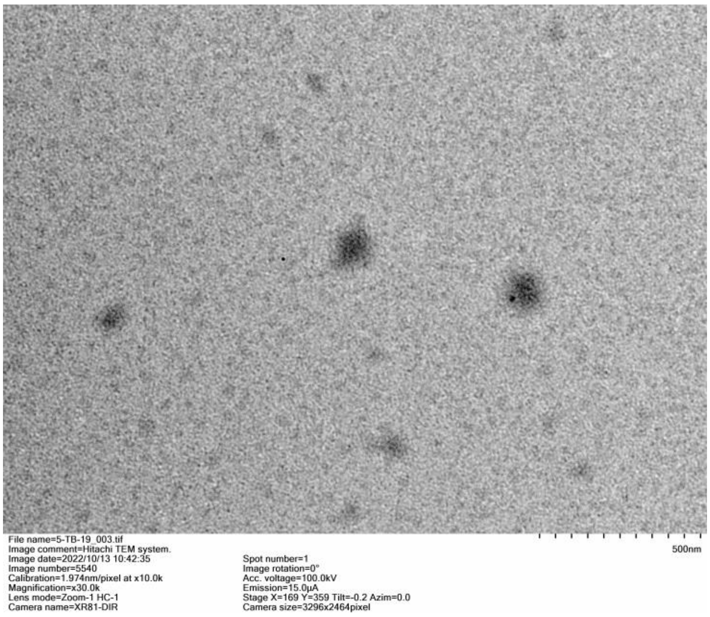

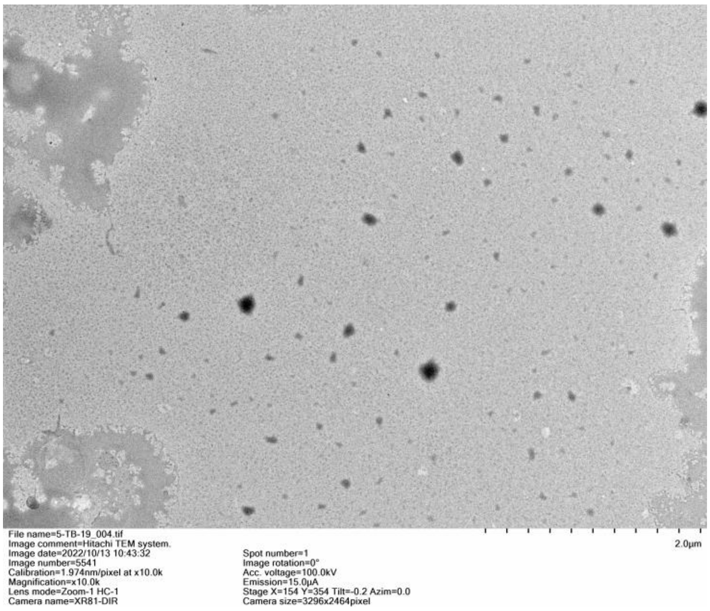

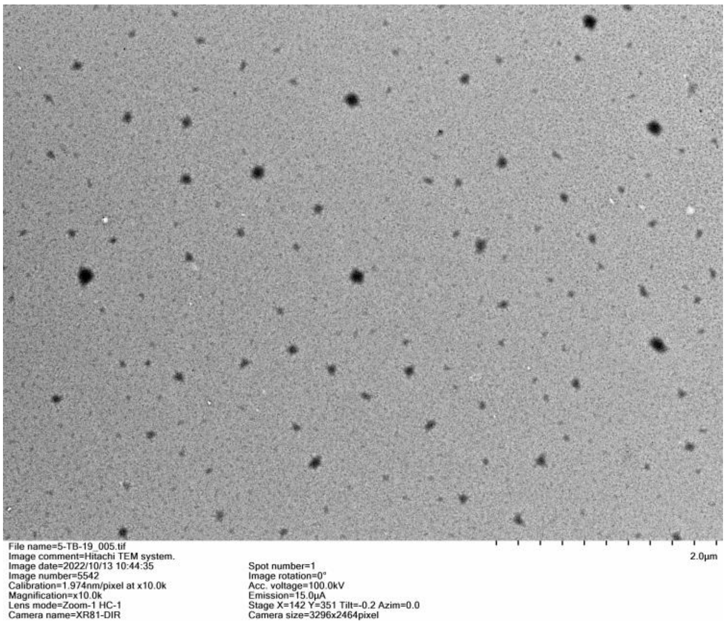

Figure S15. TEM Images of enzyme-loaded nanoreactors based on **1b**, Tris-Buffer, pH = 7.4, 25 °C.

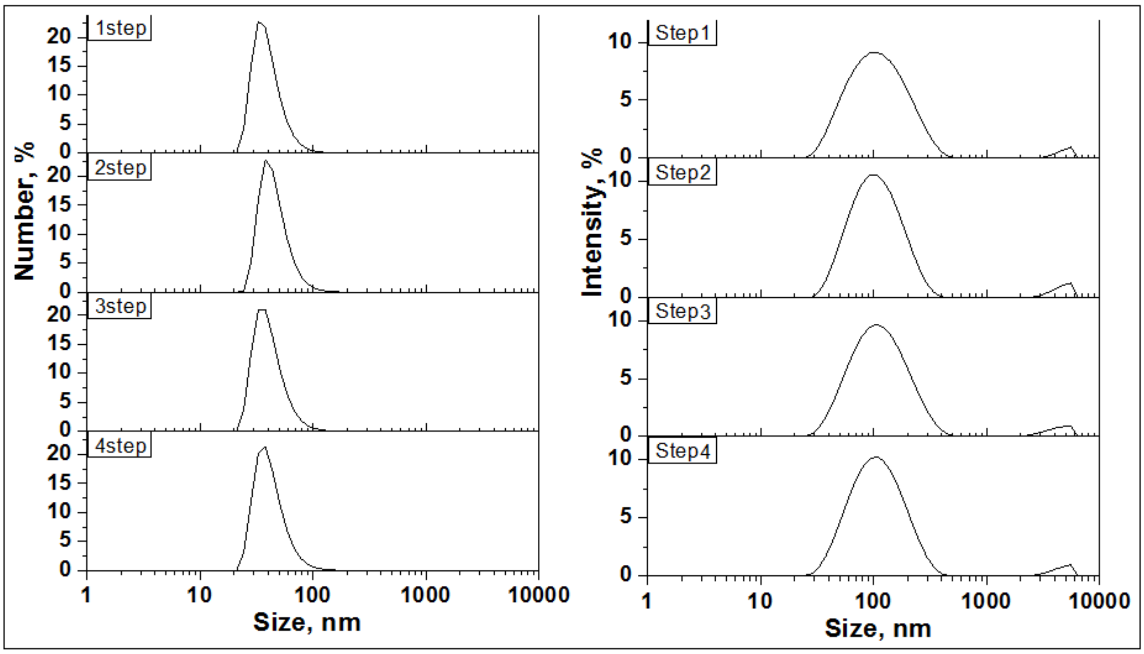

Figures S16. The size distribution using number (A-E) and intensity (F-J) parameters for nanoreators based on **1b**, copolymer concentrations 0.5%, (wt/wt), 10 mM Tris buffer, pH=7.4, 25°C, where 1 step is keeping the blood at +37°C for 1 hour, 2 step is keeping the blood at +4°C for 1 hour, 3 step – centrifuge the blood (15min, 2500rpm, +4°C), 4 step – freezing the serum at -20°C – for 7 days.

Table S1. *In vitro* stability of nanoreactors **1b** in blood.

**Sample**	**Medium**	**Z average, nm**	**PDI**
nR-PTE	Tris-buffer	95.7±0.1	0.16±0.01
1 step	blood	94±2	0.29±0.04
2 step	blood	92±2	0.27±0.03
3 step	serum	99±1	0.27±0.01
4 step	serum	92±2	0.26±0.01

where 1 step is keeping the blood at +37°C for 1 hour, 2 step is keeping the blood at +4°C for 1 hour, 3 step – centrifuge the blood (15min, 2500rpm, +4°C), 4 step – freezing the serum at -20°C – for 7 days.

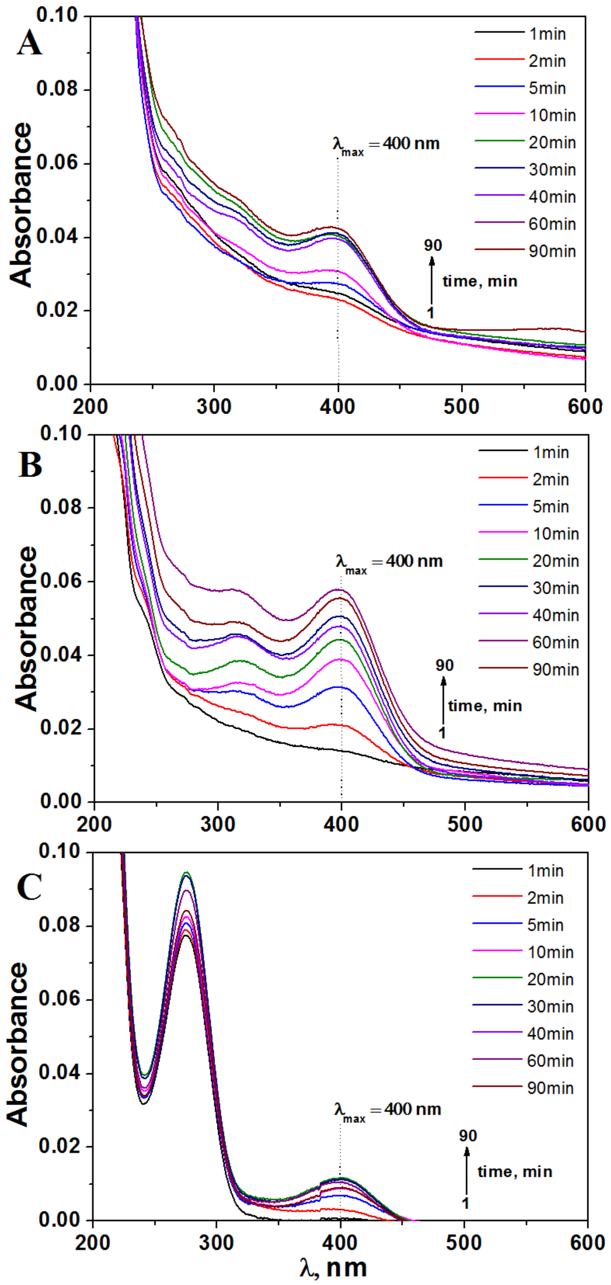

Figure S17. UV Absorbance spectra of pNp after the neutralization of POX by enzyme (A) and enzyme-loaded nanoreactors (B), control - pNp solution (pNp, 5 µM) (C), C_{POX} = 5 µM, C_{enzyme} = 1 µM, 10 mM Tris buffer, pH=7.4, 37°C.

Table S2. Prophylaxis and post-exposure treatment of paraoxon *s.c.* acute toxicity by *i.v.* administration of enzyme-loaded nanoreactors in mice, where Control 1 is the EtOH 10% in sodium chloride 0.9% solution (*s.c.*) and Control 2 is empty nanoreactor solution (*i.v.*).

Dose, mg/kg	Animals dead / total
Paraoxon acute toxicity study	
Control 1	0/3
Control 2	0/3
0.5	0/3
0.625	0/3
0.65	1/3
0.7	3/3
0.75	3/3
1.25	3/3
enzyme-loaded nanoreactors prophylactic *i.v.* administration	
5	0/3
10	1/3
15	4/6
25	3/3
enzyme-loaded nanoreactors *i.v.* treatment	
2.5	0/3
5	1/3
10	3/3

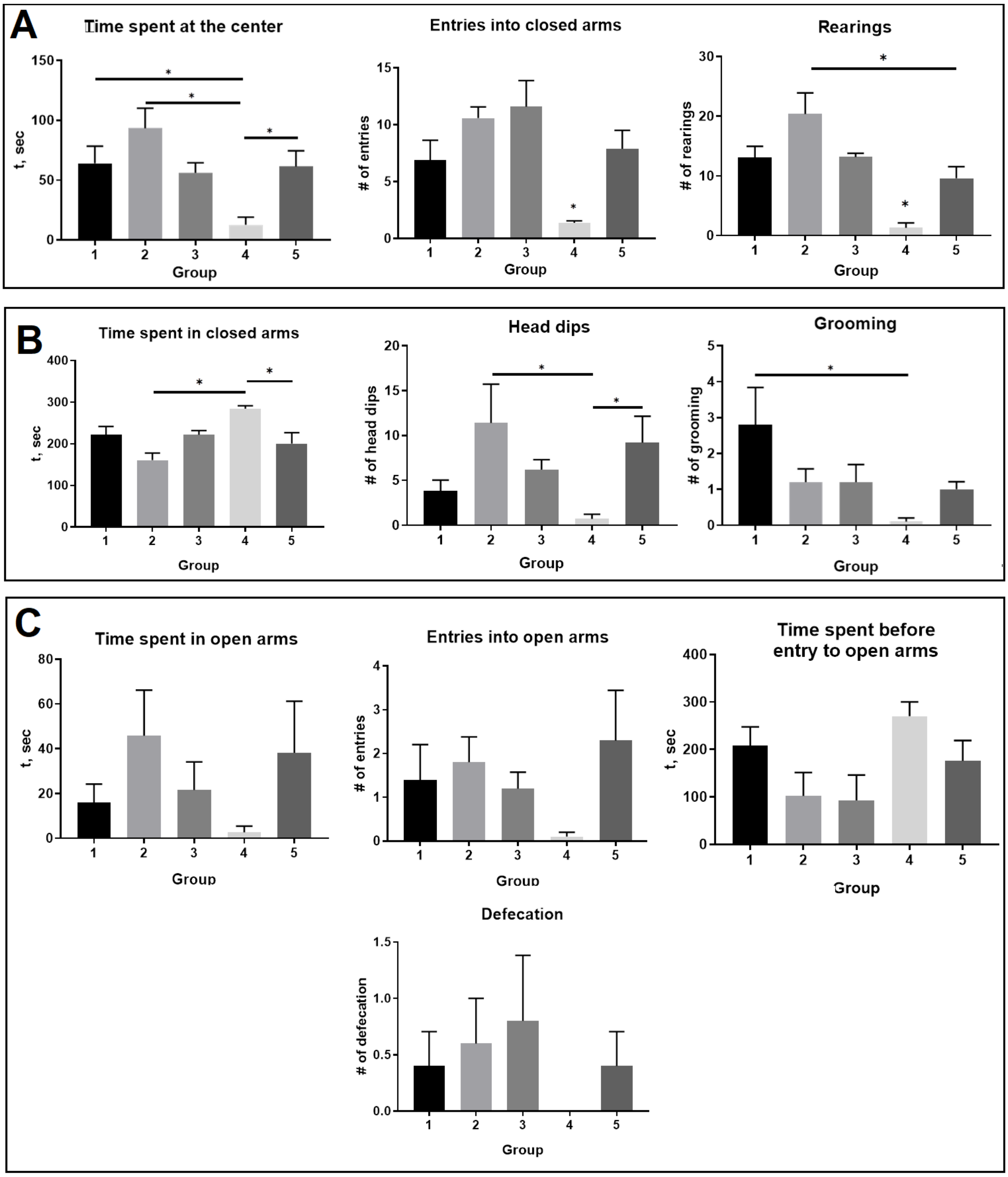

Figure S18. Elevated cross maze parameters on the 1st day of experiment, * $p < 0.05$

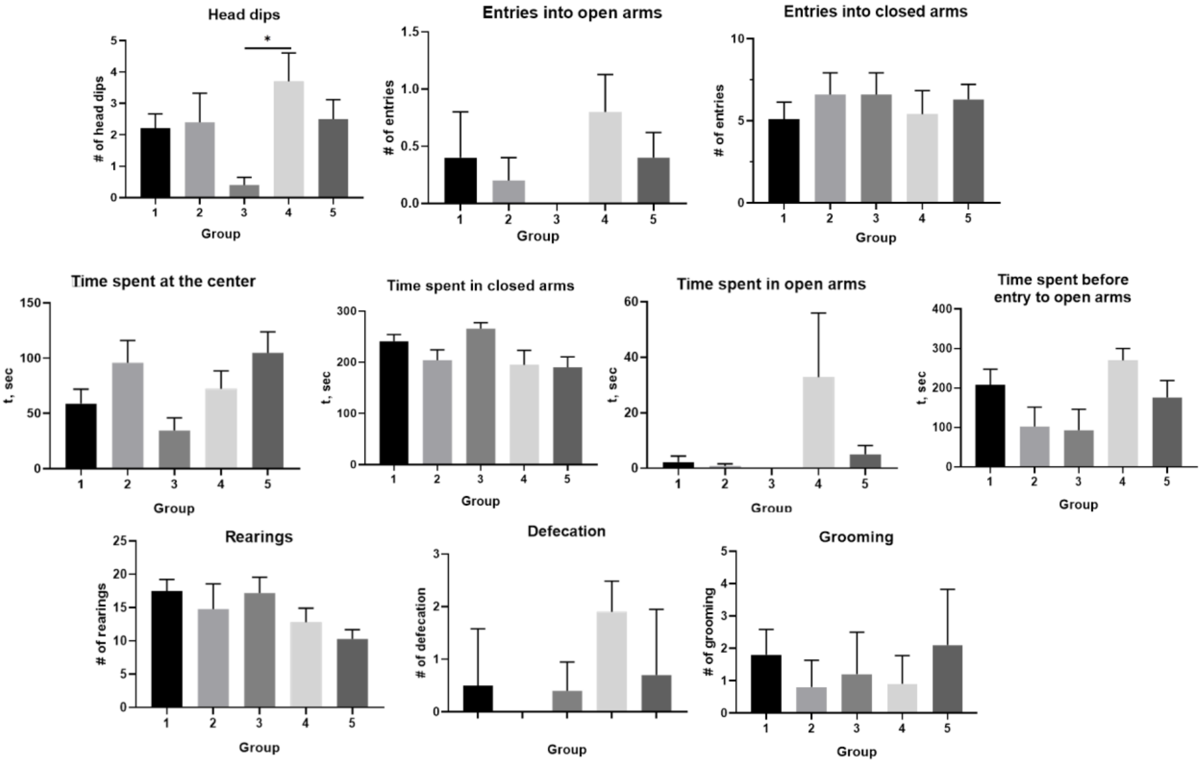

Figure S19. Elevated cross maze parameters on the 30th day of experiment, * $p < 0.05$.

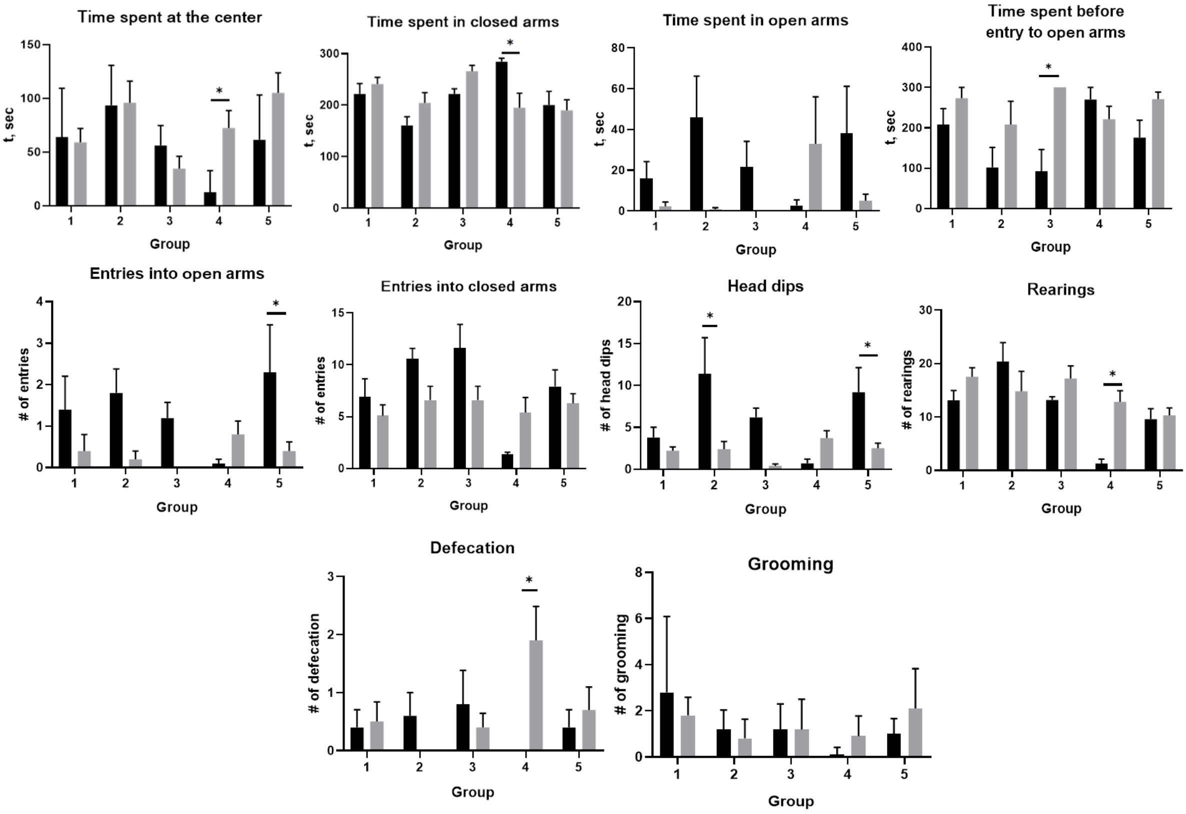

Figure S20. Elevated cross maze parameters on the 1st (black column) *vs* 30th (grey column) days of experiment, * $p < 0.05$.

Two sample t Test (16/06/23 20:31:10)

Notes

X-Function	Two sample t Test
User Name	Òàòüÿíà Ïàøèðîâà
Time	16/06/23 20:31:10

Input Data

	Data	Range
1st Data Range	[Book1]Sheet1!A	[1*:3*]
2nd Data Range	[Book1]Sheet1!B	[1*:3*]

Descriptive Statistics

		N	Mean	SD	SEM
A		3	21.96226	3.15392	1.82092
B		3	26.15995	2.83388	1.63614
	Difference		-4.19769		

t-Test Statistics

	t Statistic	DF	Prob>|t|
Equal Variance Assumed	-1.71475	4	0.16154
Equal Variance NOT Assumed	-1.71475	3.95506	0.16235

Null Hypothesis: mean1-mean2 = 0

Alternative Hypothesis: mean1-mean2 <> 0

At the 0.05 level, the difference of the population means is NOT significantly different with the test difference(0)

Figure S21. Student's t-test for free enzyme (1) and nanoreactor based on **1b** (2) 1st injection.

Two sample t Test (16/06/23 21:21:10)

Notes

X-Function	Two sample t Test
User Name	Òàòüÿíà Ïàøèðîâà
Time	16/06/23 21:21:10

Input Data

	Data	Range
1st Data Range	[Book2]Sheet1!A	[1*:3*]
2nd Data Range	[Book2]Sheet1!B	[1*:3*]

Descriptive Statistics

		N	Mean	SD	SEM
A		3	26.15995	2.83388	1.63614
B		3	25.12116	2.2397	1.29309
	Difference		1.03879		

t-Test Statistics

	t Statistic	DF	Prob>|t|
Equal Variance Assumed	0.49812	4	0.64454
Equal Variance NOT Assumed	0.49812	3.79727	0.64586

Null Hypothesis: mean1-mean2 = 0

Alternative Hypothesis: mean1-mean2 <> 0

At the 0.05 level, the difference of the population means is NOT significantly different with the test difference(0)

Figure S22. Student's t-test for nanoreactor 1st injection and nanoreactor 2nd injection (after 30 days) based on **1b**.

ANOVAOneWay (16/06/23 21:21:37)

Notes

Input Data

Descriptive Statistics

	Sample Size	Mean	Standard Deviation	SE of Mean
A	3	21.96226	3.15392	1.82092
B	3	26.15995	2.83388	1.63614
C	3	25.12116	2.2397	1.29309

One Way ANOVA

Overall ANOVA

	DF	Sum of Squares	Mean Square	F Value	Prob>F
Model	2	28.67837	14.33919	1.87079	0.23365
Error	6	45.98857	7.66476		
Total	8	74.66694			

Null Hypothesis: The means of all levels are equal.

Alternative Hypothesis: The means of one or more levels are different.

At the 0.05 level, the population means are not significantly different.

Fit Statistics

	R-Square	Coeff Var	Root MSE	Data Mean
	0.38408	0.1134	2.76853	24.41446

Figure S23. One Way ANOVA for free enzyme 1st injection, nanoreactor 1st injection and nanoreactor 2nd injection (after 30 days) based on **1b**.

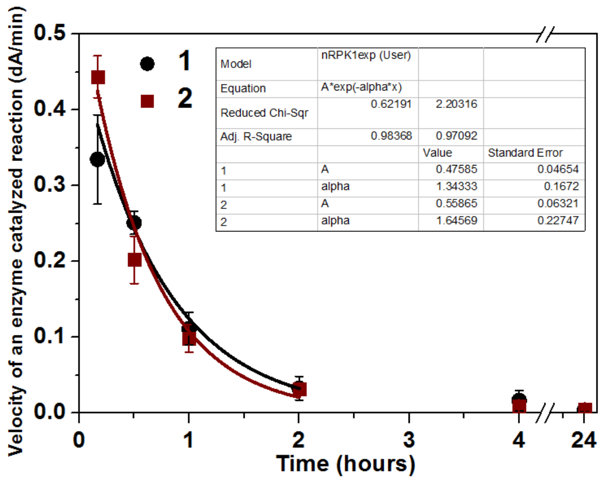

Figure S24. Pharmacokinetic profiles in mouse plasma after intravenous injection of free enzyme (1), enzyme-loaded nanoreactors made by three-block copolymers (2) after second injection (30-day). The dose of enzyme is 5.76 mg/kg. Each point represents the mean ± SD in 3 mice.

Table S3. Pharmacokinetic parameters observed in mouse after intravenous injection of free enzyme and enzyme-loaded nanoreactors second time after 1 month of 1st injections. The dose of enzyme 5.76 mg/kg. Results represent the mean ± SD for three mice, three-block copolymer nanoreactor.

Sample	number of injections	б (min^{-1})	$t_{1/2б}$* (min)
Free enzyme	2, 30-day	0.0224±0.0028	30.96±3.85
Enzyme-loaded nanoreactors	2, 30-day	0.0274±0.0038	25.27±3.49

$t_{1/2\alpha} = \ln 2/\alpha$, in which α is the distribution rate (min^{-1}) from blood. Data were analyzed via two-tailed Student’s t test ($p \leq 0.05$)

Two sample t Test (19/06/23 16:22:33)

Notes

X-Function	Two sample t Test
User Name	Òàòüÿíà Ïàøèðîâà
Time	19/06/23 16:22:33

Input Data

	Data	Range
1st Data Range	[Book1]Sheet1!1	[1*:3*]
2nd Data Range	[Book1]Sheet1!2	[1*:3*]

Descriptive Statistics

		N	Mean	SD	SEM
"1"		3	30.9595	3.85343	2.22478
"2"		3	25.27136	3.49305	2.01671
	Difference		5.68814		

t-Test Statistics

	t Statistic	DF	Prob>|t|
Equal Variance Assumed	1.89428	4	0.1311
Equal Variance NOT Assumed	1.89428	3.96205	0.13178

Null Hypothesis: mean1-mean2 = 0

Alternative Hypothesis: mean1-mean2 <> 0

At the 0.05 level, the difference of the population means is NOT significantly different with the test difference(0)

Figure S25. Student's t-test for free enzyme and three-block copolymer nanoreactor 2nd injection.

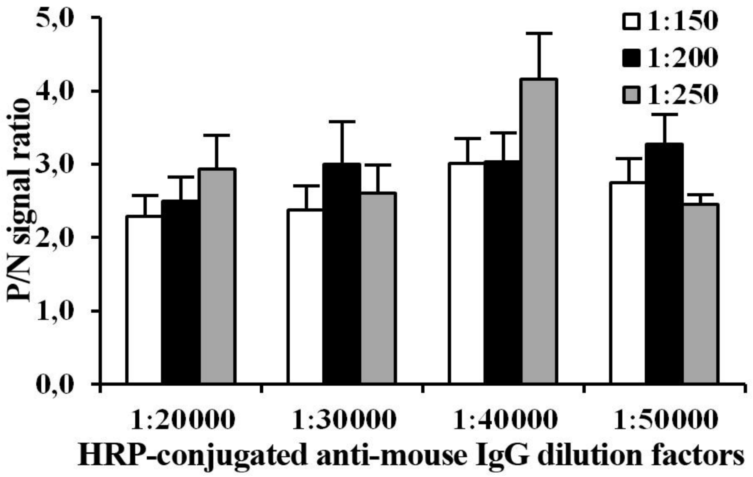

Figure S26. The ratio of the signals from positive and negative control samples (P/N signal ratio) with tested dilution factors.

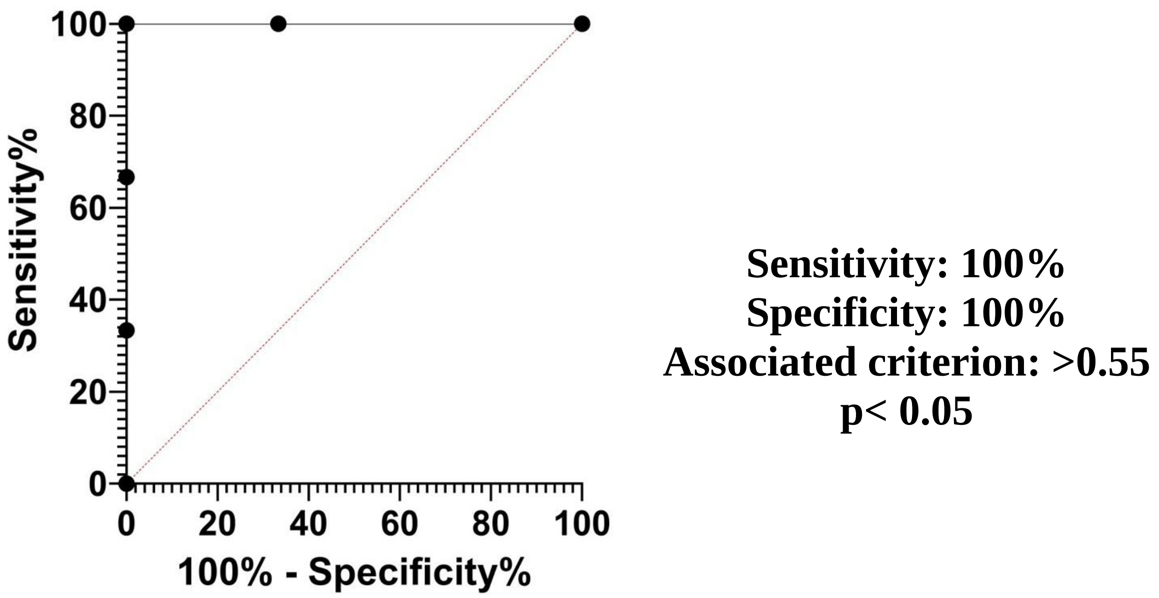

Figure S27. The receiver operating characteristic (ROC) analysis of ELISA showing sensitivity versus specificity for discrimination of positive and negative serum samples.

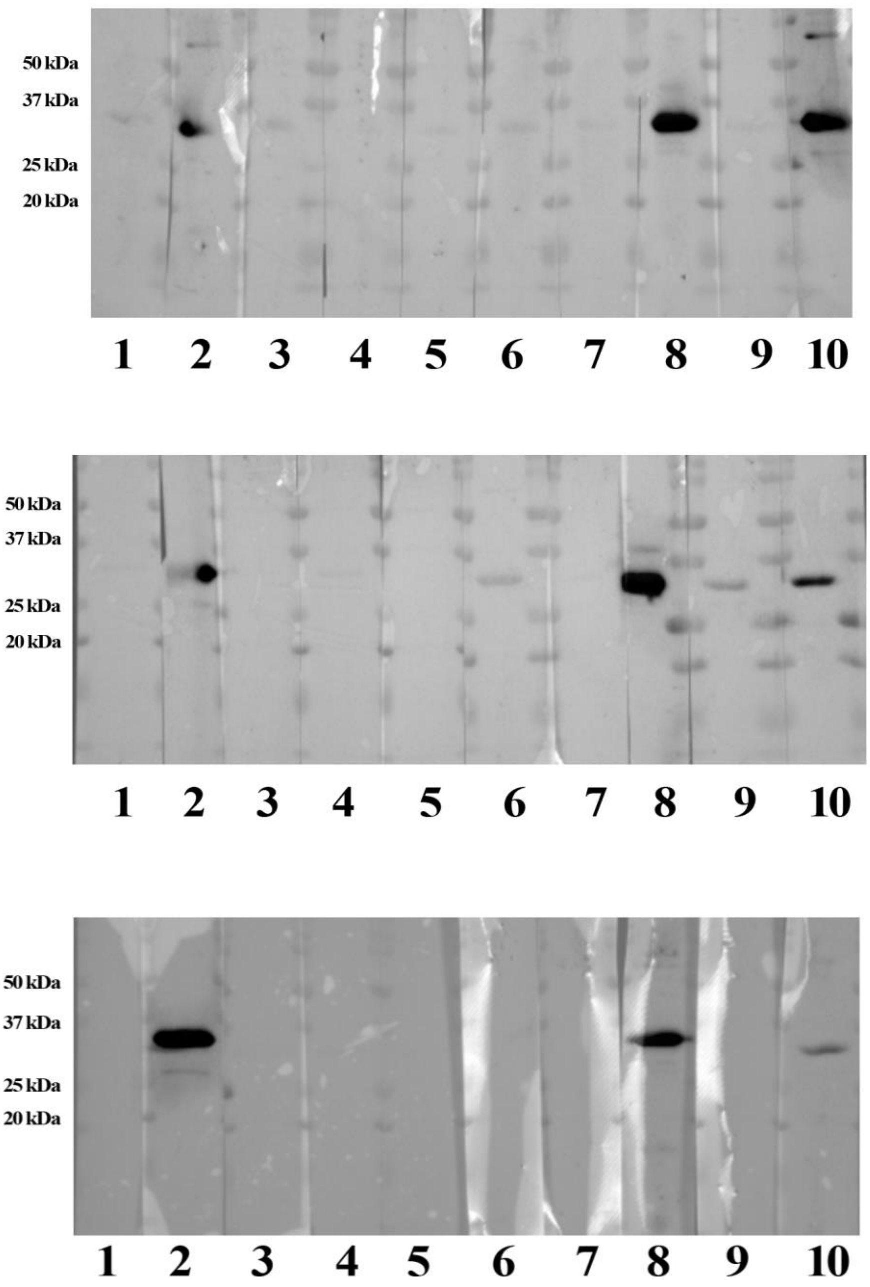

Figure S28. The western blot membranes for anti-PTE-IgG from 3 independent experiments: 1 - negative control, 2 – positive control, 3 – POX, 4 – empty nanoreactor, 5 - solvent, 6 – PTE, 7 – PTE-mPEG−PPS−mPEG nanoreactor (1 injection), 8 - PTE-mPEG−PPS−mPEG nanoreactor (2 injections), 9 - PTE-mPEG−PPS nanoreactor (1 injection), 10 - PTE-mPEG−PPS nanoreactor (2 injections).

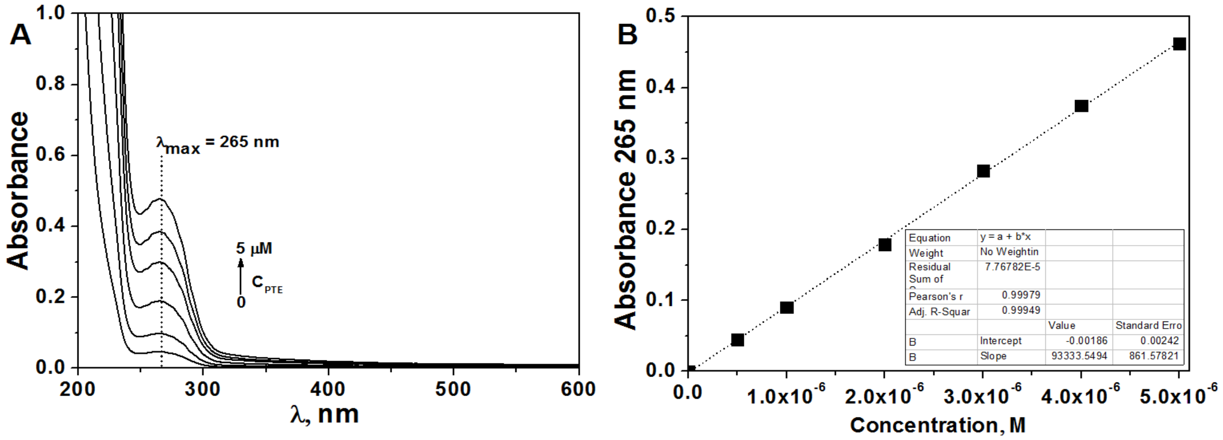

Figure S29. UV Absorbance spectra (A) and calibration curve (B) of Phosphotriesterase, 10mM Tris buffer, pH=7.4, 25°C.

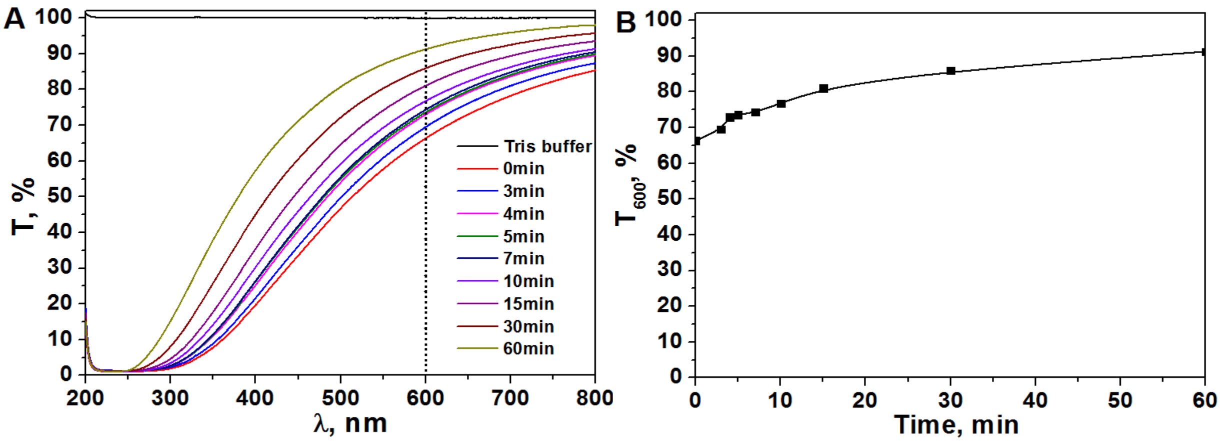

Figure S30. Transmittance of empty nanoreators under centrifugation conditions at 5000 rpm, using centrifuge over time, C_{1b} (wt/wt) = 0.5%, 10 mM Tris buffer, pH=7.4.
